# Digital Transformation and Its Relationship to the Job Performance of Employees at a Private University in Peru

**DOI:** 10.12688/f1000research.151251.1

**Published:** 2024-06-26

**Authors:** Arias Gonzales Hilda Paola, Rogger Orlando Morán Santamaría, Nikolays Pedro Lizana Guevara, Morales Salazar Pedro Otoniel, Salazar López Yasser Jackson, Yefferson Llonto Caicedo, Francisco Eduardo Cúneo Fernández, Percy Junior Castro Mejía, Milagros Judith Pérez Pérez

**Affiliations:** 1 Cesar Vallejo University, Trujillo, La Libertad, Peru; 2 Universidad Nacional Pedro Ruiz Gallo, Lambayeque, Lambayeque, Peru

**Keywords:** digital transformation, job performance, virtual education, digital tools, educational quality.

## Abstract

**Background:**

Private universities in Peru still need to implement digital transformation models to enhance the job performance of faculty and staff, achieving consistent improvement in the performance levels of university teachers by deploying technological and didactic tools for students. Therefore, the study aims to determine the relationship between digital transformation and the job performance of employees at a Private University.

**Methods:**

The research approach was quantitative, employing a non-experimental, longitudinal correlational design. The technique used was a survey, applied to a sample of 104 employees (school heads, faculty, and a director) from the university on a national level from a total population of 144.

**Results:**

Descriptive results reveal that the university has regularly adopted tools related to digital transformation. In particular, it has efficiently employed agile technologies, Big Data, and various technological means, benefiting 90% (52% and 38%) of the staff. The study also showed a high positive correlation (0.92>0.7) between digital transformation and the job performance of staff at the Private University, confirming that there is a significant connection between the variables studied.

**Conclusions:**

Therefore, creating an innovative culture across all hierarchical levels and identifying key technologies that add value to the learning flow can meet the needs of an increasingly demanding society.

## Introduction

Currently, a high percentage of companies have perceived the need to create innovative models due to the COVID-19 pandemic, with the intent to add value to the end consumer and ensure business continuity ( Marchesani et al., 2023; Acciarini et al., 2023). Consequently, the educational sector cannot escape this transformation, as it is a highly attractive sector that needs to be at the forefront of tools and digital channels to compete and offer professional profiles that meet the demands of the job market and successfully ensure their integration; at this stage of the study, the goal is for teachers to utilize technology and educational applications to enhance attention and teaching improvement ( Kang & Park, 2023; Valdés & Gutiérrez, 2023; Chigbu et al., 2023).

An innovative business model allows for the adoption of technology to efficiently offer products or services, optimizing costs and time, making it more sustainable and responsive in the long term to challenges posed by external forces ( Rojas et al., 2024; Afawubo & Noglo, 2022). Under this context, the business environment has drastically transformed, leading to the adoption of agile technologies that enable the continued competitive performance within the sector ( Shahiduzzaman & Kowalkiewicz, 2018).

Digital transformation in Peru is an ongoing process of cultural change supported by the intensive use of digital technologies, systematization, and data analysis to generate economic, social, and value impacts for individuals ( Aurazo & Vega, 2021; Gupta et al., 2020). It is important to note that the Secretary of Government and Digital Transformation of the Presidency of the Council of Ministers (PCM) leads the coordination, implementation, and evaluation of the National Digital Transformation Policy, as well as its strategies, policies, plans, regulations, directives, projects, and corresponding platforms ( Single Digital Platform of the Peruvian State, 2023). Therefore, private companies are acting in relation to the four main key aspects: strategy, customer interaction, business model transformation, and digital talent acquisition; on the other hand, in the public sector, the lack of organizational continuity prevents coordinated action in these areas ( ESAN, 2022; BID, 2023).

On the other hand, universities are a main part of economic development globally, which is why many Latin American countries have adopted virtual reality as a key element to foster innovation and provide educational quality accessible to all, achieving social inclusion in the process ( Maliqueo et al., 2021). According to UNESCO (2018), the job performance of teachers forms a solid and influential part of the journey to quality education. Thus, adopting digital transformation facilitates a quick response to the constant ups and downs posed by the market; however, it requires a review and change in the company culture, its operations, and a shift to agile technologies with the aim to replace and improve existing resources ( Delgado, 2020).

It is important to note that the application of digital tools must be managed by specialists, as such technological products must serve a specific purpose and not be adopted merely for appearing innovative ( Bindra, 2018). For this reason, companies view technology as a tool that encourages the creation of current models ( Mamabu, 2017) and allows facing challenges posed by the market ( Valderrama, 2019). In this regard, education will have a better response if implemented correctly in the digitalization processes, thereby improving productivity ( Musaxonovna, 2022). At this point, Nwaiwu (2018) stated that education should not be limited to a physical space but rather promote the breakdown of barriers, giving teachers and students new roles in which they can interact virtually effectively and securely. For Ledahawsky (2022), digital transformation is a business opportunity as it allows the development of a technological vision in companies.

With this in mind, there is a need to categorize digital transformation as a process that must be prioritized in all organizations and not as an option for using digital tools. Likewise, the search for innovative business models that make a difference from conventional industries is evident ( Bindra, 2018; Amarista, 2005). With this, it is evident that a large number of companies around the world use digital transformation as a means to be categorized as “digital companies,” hence the importance of adopting technology at all business levels.

As revealed, the research seeks to optimize the proper job performance of teachers, as they are the direct channel with the end consumers, who at this point are the students, through the use of digital transformation, thus ensuring the improvement of economic and social conditions. In this way, it seeks to reduce the gaps of inequality in quality education nationwide. For this reason, the general objective is to determine the relationship between digital transformation and the job performance of employees at a Private University.

## Theoretical framework

To deepen the foundation of the literature review for this study, a review of scientific articles was conducted based on two relevant areas: a) digital transformation and b) job performance. Initially, international scientific articles were considered; Nunes & Malagri (2023) provided a documentary review in Brazil, setting key parameters for student development in a modern and changing society. Following this, Bazzo et al. (2022) through their correlational study with a qualitative focus involving 104 participants highlighted the lack of digital tools and the poor performance of teachers, illustrating the impact of technology on teachers' job performance. Furthermore, Granda & Bermeo (2022) in their empirical and analytical study emphasized the importance of mindset change in organizational culture to adapt technologies in business process management. Townsend & Figueroa (2022) in their correlational-inductive article evaluated the quality of digital transformation on methods and labor productivity. De Matos et al. (2020), in their exploratory-qualitative study of 68 teachers at a Brazilian university, demonstrated that education in Brazil requires technologies for better student performance. Similarly, Cuenca et al. (2020) in their correlational study conducted at seven educational institutions in Spain showed that digital transformation is influenced by organizational climate, leadership, and employee profiles.

Nationally, Huamán & Medina (2022) in their correlational descriptive study showed that closing gaps in communication and response are inherent to the use of digital transformation. Collantes & Collantes (2022), in their correlational study involving 70 employees from the government sector, found a direct relationship between public policies and the use of digital transformation tools. Laurente (2021) in his documentary review of 54 technology sector companies demonstrated that digital transformation improves productive processes and fosters a culture based on technology and innovation. Moreover, Tippe & Soto (2021) in their hermeneutic-heuristic method study with 50 teachers from an institution argued that the government has the task of closing inequality gaps so that through internet access and various technologies, a quality education accessible to all Peruvians can be achieved. Guzmán et al. (2020), in their explanatory-deductive study at four Peruvian educational institutions, demonstrated the implications of digital transformation on customer-oriented attention and the performance of assigned tasks. Lastly, Ramirez & López (2018) highlighted the importance of converting traditional educational enterprises into purely virtual ones, stating that in the long term, this promotes better teaching performance and better response to market demands.

Regarding the study variables, we have

A) Digital Transformation
  Regarding the theoretical bases, we define digital transformation as the digital capability applied to internal processes to increase efficiency. Regarding the theoretical foundations of digital transformation, according to Matt et al. (2015), the term digital transformation evolves from the concept of “digitization,” that is, processes that support business transformation. Ramirez & López (2018) stated that digital transformation “is a model that focuses on the digital approach as a strategy that identifies the value proposition of the organization, considering having elements to innovate and respond to market demands and analyses. Ultimately, it seeks to concentrate a correct operational structure in order to achieve and maintain excellent processes.” (p.12). For Cueva (2020), the term digital transformation refers to all digital technology adopted by organizations to improve the performance and capabilities of the employees; Alonso (2017) described it as the induction of all technology for the improvement of internal processes in companies.
B) Job Performance
  According to theories related to the variable of job performance, these are based on a procedure that evaluates, measures, and influences human behavior, generating attributes and outcomes related to work. Regarding the foundational theories of job performance, according to Klehe & Anderson (2007), this theory involves the specific ability required by the position, the level of effort required and demonstrated, and the proper application of discipline and moral values (avoiding counterproductive behaviors), ultimately these core aspects could apply to any position. Additionally, according to the adaptive performance theory, performance has defined the quality of work performed by employees within a company. Mamani & Cáceres (2019) suggested that job performance is about achieving objectives through strategies accompanied by patterns of behavior, competencies, and skills of the workers; Bellot (2011) indicated that it is important to measure job performance as it provides greater competitiveness and productivity among employees. Thus, it is defined as any skill or ability of the worker that leads to an efficient outcome.

## Methods

### Type, design, and scope

The methodology of the article was developed under a methodological process called “Quantitative Approach,” as it involved statistical analysis of the selected sample from the study object. Furthermore, the research scope is of an applied, quantitative, correlational, and non-experimental type ( Maxwell & Reybold, 2015; Aarsman et al., 2024). Non-experimental, as the behavior of both variables was not altered; in other words, only the variables were observed under a natural context. Correlational scope, because the purpose of the research was to find relationships between the variables in question.

### Population and sample

The population consisted of 144 employees, with a sample size of 104 employees ( Otzen & Manterola, 2017; Stratton, 2021), including managers, directors, and teachers from the Professional Business School at the following campuses: Lima Este, Lima Norte, Chiclayo, Trujillo, Ate, Callao, and Piura. Below is the distribution of the sample across branches:

**Table 1.** Characteristics of the sample by branch.

		Frecuency	Percentage	Valid Percentage	Accumulated Percentage 3/3
Valid	Lima Norte	27	26.0	26.0	26.0
	Callao	10	9.6	9.6	35.6
	Chiclayo	12	11.5	11.5	47.1
	Lima Este	15	14.4	14.4	61.5
	Piura	23	22.1	22.1	83.6
	Ate Vitarte	14	13.5	13.5	97.1
	Trujillo	3	2.9	2.9	100.0
	Total	104	100.0	100.0	

*Note:* The table displays the distribution of teachers by branch nationwide *.*

For sample selection, various eligibility criteria were employed. Inclusion criteria included employees with more than 6 months of tenure in the organization and employees with subordinates. Additionally, employees of both genders, regardless of age, were considered. Finally, exclusion criteria did not include employees whose tenure in the organization is less than 6 months (new hires), thus ensuring a more homogeneous learning curve for the activities.

### Procedure

According to the research design, it follows rational or logical and systematic steps, which facilitated the resolution of the research problem. The most important steps of the research included the following points: conception of the idea, problem statement, research design, sample selection, data collection, data analysis, data interpretation, and discussion of results.

### Techniques and data collection instruments

It is important to note that the technique applied, considering the dimensions and indicators of the study, is the survey, which was developed through a questionnaire. Regarding the Digital Transformation variable, 32 questions were considered (with 5 options structured on a Likert scale), while for the Work Performance variable, 26 questions were considered (also with 5 options structured on a Likert scale). It is worth mentioning that the survey was administered to the sample, and interviews were conducted with school heads for greater accuracy in the results ( Story & Tait, 2019; Vaske, 2019).

### Validity and reliability

The validity of the survey instrument was established through the informed opinion of individuals with extensive experience and legitimacy to provide information, testing, and judgment. Additionally, the reliability of the instrument was assessed through the Cronbach's Alpha coefficient, where the questionnaire for digital transformation obtained a score of 0.973 and for work performance, 0.976, both considered highly reliable ( Kimberlin & Winterstein, 2008; Wyse et al., 2016).

### Data analysis

Data analysis was conducted using IBM SPSS Statistics 26 software. This has allowed for efficient descriptive results of each variable, enabling the testing of hypotheses proposed in the research. In the results obtained from the “Normality Test,” the Kolmogorov-Smirnov test was employed, as the sample size exceeded 104 participants. The non-parametric Spearman's Rho correlation coefficient was also used, as the significance level is considered less than 0.05 ( Bryman, & Cramer, 2011; Pallant, 2020; Saiyidi & Hadrian, 2024).

### Ethical component

During the research process, the participation of the 104 selected participants was obtained, who provided authorization and consent to obtain timely information for the development of the research. Considering the general principles governing ethics in scientific research, including intellectual honesty, truthfulness, transparency, human integrity, respect for intellectual property, justice, and responsibility, the study aims to comply with “The Code of Ethics in Research of César Vallejo University, version 01; through University Council Resolution No. 0340-2021-UCV”.

## Results

The study aimed to determine the relationship between digital transformation and the job performance of employees at a University. A quantitative study was conducted based on the survey data; according to the digital transformation variable, the following was observed:

**Table 2.** Frequencies and percentages for the Digital Transformation variable and its dimensions.

Levels	Digital transformation		Process		Organizational culture		Strategic vision		Business model	
	F	%	F	%	F	%	F	%	F	%
Good	40	38%	30	29%	34	33%	34	33%	52	50%
Fair	47	45%	51	49%	54	52%	52	50%	28	27%
Poor	17	16%	23	22%	16	15%	18	17%	24	23%
TOTAL	104	100%	104	100%	104	100%	104	100%	104	100%

*Note.* Instrument (questionnaire) applied to the sample of the Private University, 2023. F is Frequency and % is Percentage.

According to Table 2, the percentage of the independent variable and its dimensions regarding satisfaction levels related to processes, organizational culture, strategic vision, and business model are as follows: for the digital transformation variable, 38% good level, 45% regular level, and 16% poor level. For the process dimension, 29% good, 49% regular, and 22% poor. For the organizational culture dimension, 33% good, 52% regular, and 15% poor. For the strategic vision dimension, 33% good, 50% regular, and 17% poor. Lastly, for the business model dimension, 50% good, 27% regular, and 23% poor. Teachers believe that the university is not investing correctly in agile technologies for better performance, as the use of technology would offer better and correct decision-making and a flexible response to external events. Furthermore, it is evident that the digital channels and tools being applied by the university are not significantly impacting educational management, necessitating improvements and providing tools that change or modify the current business model and foster a culture of innovation.

**Figure 1.**
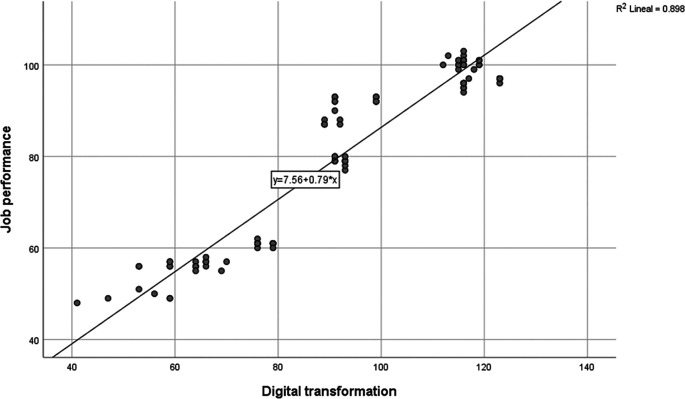
Scatter Plot. *Note:* Prepared based on questionnaire data. R2 is the coefficient of determination.

**Table 3.** Frequencies and percentages for the variable Work performance and its dimensions.

Levels	Work performance		Personality		Productivity		Learning		Perception	
	F	%	F	%	F	%	F	%	F	%
Good	64	62%	46	44%	52	50%	59	57%	52	50%
Fair	34	33%	34	33%	14	13%	45	43%	21	20%
Poor	6	6%	24	23%	38	37%	0	0%	31	30%
TOTAL	104	100%	104	100%	104	100%	104	100%	104	100%

*Note.* Instrument (questionnaire) applied to the sample of the Private University at the National level, 2023.

In Table 3, the percentage of the dependent variable and its dimensions regarding satisfaction levels related to the influence of personality, productivity, learning, and perception are: for the job performance variable, 62% good level, 33% regular level, and 6% poor level. For the personality dimension, 44% good, 33% regular, and 23% poor. For the productivity dimension, 50% good, 13% regular, and 37% poor. For the learning dimension, 57% good, 43% regular, and 0% poor. Finally, for the perception dimension, 50% good, 20% regular, and 30% poor. It is evident that the university does not influence the motivation of teachers, and the existence of job pressure generates stress events, thereby reducing performance. Teachers also consider the task demands excessive relative to their available time, adding to the authoritarianism of school heads. However, it was also found that teachers possess the necessary skills and competencies to meet demands, provided the weaknesses are addressed.

**Table 4.** Normality test using Kolmogorov-Smirnov.

	Kolmogorov-Smirnov		
	Statistic	gl	Sig.
Digital transformation	.166	104	.000
Processes	.157	104	.000
Organizational culture	.161	104	.000
Strategic vision	.160	104	.000
Business model	.210	104	.000
Work performance	.197	104	.000
Personality	.178	104	.000
Productivity	.325	104	.000
Learning	.165	104	.000
Perception	.217	104	.000

*Note:* The table presents the normality test according to Kolmogorov-Smirnov applied to the variables and their dimensions.

The hypothesis testing was performed using Pearson's correlation, which, as shown in Table 5, indicates a significant relationship between the study variables, as the observed significance level (bilateral sig.) is 0.001; being less than 0.05, the alternative hypothesis is rejected.

As per Table 4, the Kolmogorov-Smirnov test for normality is conducted as the sample size exceeds 50, aiming to observe the behavior of the variables and their distribution, concluding at this point that the distribution is not normal. Subsequently, it was analyzed whether there is a correlation between the components, through a scatter plot with Job Performance on the ordinate axis and Digital Transformation on the abscissa axis, as illustrated below.

A higher level of digital transformation corresponds to better job performance, interpreting that the behavior of digital transformation explains 89% of the behavior of job performance; in its linear regression equation, there is a 79% likelihood that a higher propensity for digital transformation (variable x) generates better job performance (variable y). Therefore, Spearman's Rho needs to be calculated, as the study variables are ordinal (see Table 5 and Figure 1).

**Table 5.** Spearman's Rho correlation between the variables of digital transformation and work performance.

			TOTAL_Digital_Transformation	TOTAL_Work_Performance
Spearman's Rho	TOTAL_Transformacion_digital	Correlation coefficient	1.000	.925 * *
		Sig. (bilateral)	.	.000
		N	104	104
	TOTAL_Work_Performance	Correlation coefficient	.925 * *	1.000
		Sig. (bilateral)	.000	.
		N	104	104

Note. *Rho Spearman.* r=0.095 y p=0.340

* The correlation is significant at the 0.01 level (two-tailed).

A high, statistically significant positive correlation is shown (0.92>0.7), rejecting the null hypothesis, thereby accepting the alternative hypothesis (There is a significant relationship between digital transformation and the job performance of employees at a Private University, 2023), indicating a significant relationship between the study variables.

## Discussion - Conclusions – Recommendations

Descriptive results suggest that the university implements digital transformation tools moderately, effectively utilizing agile technologies, BigData, and technological applications or media for 90% (52% and 38%) of employees. According to Granda & Bermeo (2022), a continuous change through the implementation of innovative and agile technologies is envisioned for optimal process improvement. It is concluded that processes are automated through platforms and tools aimed at reducing or optimizing time through the same.

On the other hand, job performance is affected by the organization's motivation for high performance. While there is existing pressure to achieve results, this must be managed along with its tolerance, as over 90% of teachers contend that these demands provide a sense of urgency, but in many cases, they exceed the attitudes of the employee. The organization must address the deficiencies in stress management and motivation and how a job under pressure can dramatically diminish individual and group performance. Thus, Taghipour & Dejban (2013) reaffirms our refined critique that job performance consists of the motivation that the worker has to perform the function.

Finally, the research recommends redefining the university's business model, creating an innovative culture at all hierarchical levels, and identifying key technologies that add value to the learning flow. Additionally, feedback on the application of the same is important, and if necessary, training sessions should be coordinated with the teacher to ensure that the information provided is applied correctly. Equally important, a subsequent study on leadership is necessary, as the information received indicates poor practices by authorities that prevent teachers from responding adequately or feeling motivated in their daily tasks and/or duties.

### Ethics and consent

During the research process, 104 selected participants from the population took part. The procedures for obtaining results began with an email invitation to each participant, receiving their informed consent in writing via email. Additionally, each participant was provided with a detailed description of the study, its objectives, and the procedures involved. Data collection employed various digital strategies, notably live meetings via the Zoom platform and Google Forms, ensuring the confidentiality of responses for the researchers at all times. “The development of the research article was based on the guidelines established in the “Code of Ethics in Research of the Universidad César Vallejo, version 01”; by Resolution of the University Council N° 0340-2021-UCV, which was approved on 19 July 2022.

Reporting guidelines: 10.5281/zenodo.11322516

Creative Commons Zero v1.0 Universal (CC0 License)

## Data Availability

Zenodo. Evaluation of the administrative management model in a municipality in Peru, incorporating the Intelligent Organization Theory. 10.5281/zenodo.11322516 ( Arias et al., 2024). This project contains the following underlying data:
• Data SPSS.sav • Rectoral Resolution N° 760-2007_UCV_CODE OF ETHICS.pdf This project contains the following extended data:
•
Figure 1_Scatter Illustration.png • Informed Consent.pdf • Instrument_F1000.pdf Creative Commons Zero v1.0 Universal (CC0 License)
