# Supplementary material for: Digital Transformation and Its Relationship to the Job Performance of Employees at a Private University in Peru
Source: F1000Res. 2024 Jun 26;13:692. [Version 1] doi: 10.12688/f1000research.151251.1 (PMC11422760; doi:10.12688/f1000research.151251.1)
Supplement: Peer review history

# Reviewer report: Reviewer response for version 1

**DOI:** 10.5256/f1000research.165884.r316917

**Published:** 2024-09-24

**Authors:** Ricardo Fernando Cosio Borda

**Affiliations:** 1 Universidad EAN, Bogotá, Bogota, Colombia

In general, the article complies in substance and form. It presents a review of relevant literature, based on current sources and essentially on scientific articles that contribute to a solid theoretical framework.

 The methodology has been clearly detailed and supported by statistical elements, essentially the validation and reliability of the instrument used for data collection. The conclusions are relevant according to the objective of the study and at a thematic level it is a quite pertinent contribution.

 As an opportunity for improvement for future research, the object of study can be broader and not only applied to a single institution; it could be of greater contribution to carry out a similar study but considering different institutions in emerging countries.

Is the work clearly and accurately presented and does it cite the current literature?

Yes

If applicable, is the statistical analysis and its interpretation appropriate?

Yes

Are all the source data underlying the results available to ensure full reproducibility?

Yes

Is the study design appropriate and is the work technically sound?

Yes

Are the conclusions drawn adequately supported by the results?

Yes

Are sufficient details of methods and analysis provided to allow replication by others?

Yes

Reviewer Expertise:

International business, management, sustainability and economy.

I confirm that I have read this submission and believe that I have an appropriate level of expertise to confirm that it is of an acceptable scientific standard.

# Reviewer report: Reviewer response for version 1

**DOI:** 10.5256/f1000research.165884.r296617

**Published:** 2024-07-09

**Authors:** James Sunney Quaicoe

**Affiliations:** 1 Tallinn University, Tallinn, Estonia

The paper reports on a study that explored the relationship between digital transformation and job performance in higher education institutions. 

 The authors set the stage for the article by presenting a very well-composed introduction section. 

 The instruction is made up of relevant literature that defines the background of the research. 

 The paper presents a clear and concise theoretical underpinning of the study, providing a solid framework for the research, though skeletal, enough to situate the primary focus  - i) digital transformation and ii) job performance into the research structure.

 The paper meticulously addresses the study's methodology, including the sample, the instruments, the data collection procedure, and the analysis. Further steps were taken to test the instrument's validity and reliability, instilling confidence in the study's rigor. The authors also reported on steps taken to address the study's ethical requirements.

 The results establish the insightful correlations between digital transformation and job performance.

 Overall assessment, the research is topical, and the paper is well written. The language used in the article is scholarly. The paper can be indexed.

Is the work clearly and accurately presented and does it cite the current literature?

Yes

If applicable, is the statistical analysis and its interpretation appropriate?

Yes

Are all the source data underlying the results available to ensure full reproducibility?

Partly

Is the study design appropriate and is the work technically sound?

Yes

Are the conclusions drawn adequately supported by the results?

Yes

Are sufficient details of methods and analysis provided to allow replication by others?

Yes

Reviewer Expertise:

Educational leadership and administration; digital transformation and lifelong learning; institutional-based digital transformation and padegogical innovation in schools; digital lteracy development  and digital divide interventions.

I confirm that I have read this submission and believe that I have an appropriate level of expertise to confirm that it is of an acceptable scientific standard.
